# A review of Cyclidiinae from China (Lepidoptera, Drepanidae)

**DOI:** 10.3897/zookeys.553.6153

**Published:** 2016-01-14

**Authors:** Nan Jiang, Shuxian Liu, Dayong Xue, Hongxiang Han

**Affiliations:** 1 Key Laboratory of Zoological Systematics and Evolution, Institute of Zoology, Chinese Academy of Sciences, Beijing 100101, China; 2 University of the Chinese Academy of Sciences, Beijing 100049, Chin

**Keywords:** DNA barcodes, morphology, new subspecies, new synonymy, taxonomy

## Abstract

The subfamily Cyclidiinae from China is reviewed: two genera and seven species are reported from China. One new subspecies, *Cyclidia fractifasciata indistincta* **subsp. n.**, is described. Two new synonyms are established: *Cyclidia substigmaria* (Hübner, 1831) (= *Cyclidia substigmaria brunna* Chu & Wang, 1987, **syn. n.** = *Cyclidia tetraspota* Chu & Wang, 1987, **syn. n.**). One misidentification in Chu & Wang (1987) is corrected. Identification keys and diagnoses for all discussed Chinese species are provided. External features and genitalia are depicted. In addition, results of DNA barcoding for five taxa of *Cyclidia* are briefly discussed.

## Introduction

The subfamily Cyclidiinae Warren, 1922, is the smallest subfamily within four subfamilies (besides Drepaninae, Oretinae, and Thyatirinae) of Drepanidae. This subfamily was first proposed as Eucherinae by Strand (1911) based on the genus *Euchera* Hübner, 1825. Later, it was treated as a separate family (Inoue 1962), followed by other authors (Fletcher 1979, Chu and Wang (1987, 1991) (Yan et al. 2009). However, Minet (1983) regarded Cyclidiinae as a subfamily of Drepanidae, based on the study of the tympanal organs. This treatment was later followed by many researchers, e.g. Holloway (1998) and Minet (2002), and was also supported by molecular data (Wu et al. 2010).

Recently, Chen (2011) performed a phylogenetic analysis of Cyclidiinae, based on morphological characters. In his study, the monophyly of respectively Cyclidiinae, *Cyclidia* and *Mimozethes* was supported, and most synapomorphies for Cyclidiinae proposed by previous studies were shown to be plesiomorphies. Three major synapomorphies of Cyclidiinae were given (Chen 2011): 1) the developed anterotergal syndeses (a paired of semi-translucent structure, see Yan et al. 2005) at the anterior margin of the 2^nd^ tergum; 2) a pair of androconial hair-pencils on the 2^nd^ pleuron of the male and 3) the short and robust gnathos in the male genitalia.

Species of Cyclidiinae are distributed in the Palearctic Asia and Oriental region. Up to the present, two genera (*Cyclidia* Guenée, 1858 and *Mimozethes* Warren, 1901) have been recognized in Cyclidiinae. Ten species and eight subspecies are included in *Cyclidia*, with six species and four subspecies (*Cyclidia substigmaria substigmaria* (Hübner, 1831), *Cyclidia substigmaria brunna* Chu & Wang, 1987, *Cyclidia substigmaria intermedia* Prout, 1918, *Cyclidia tetraspota* Chu & Wang, 1987, *Cyclidia rectificata rectificata* (Walker, 1862a), *Cyclidia fractifasciata* (Leech, 1898), *Cyclidia sericea* Warren, 1922, *Cyclidia orciferaria* Walker, 1860) recorded in China (Moore 1886, Aurivillius 1894, Swinhoe 1899, Strand 1911, Warren 1914, Bryk 1943, Inoue 1962, Chu and Wang 1991, Chang 1989, Holloway 1998, Lutz and Kobes 2002). Three species are included in *Mimozethes*, with two species recorded in China, *Mimozethes lilacinaria* (Leech, 1897) and *Mimozethes angula* Chu & Wang, 1987. However, the taxonomy of some Chinese taxa remained unclear (e.g. the subspecies delimitation of *Cyclidia substigmaria*; the taxonomic status of *Cyclidia tetraspota* and the puzzling distribution of *Cyclidia sericea*) (Yan et al. 2009, Chen 2011). It is obviously that further research is needed and molecular markers could be used to clarify these problems.

The DNA barcoding method using a 658 bp base pair fragment of the cytochrome c oxidase subunit I gene (COI) as a tool for species discrimination was first put forward based on two hundred closely related species of Lepidoptera (Hebert et al. 2003). It has since been successfully used for species delimitation in lepidopteran species that are difficult to separate morphologically (see Hajibabaei et al. 2006, Yang et al. 2012). The barcoding gap between intra- and inter-specific variation was used for species discrimination (Hebert et al. 2004a, Meier et al. 2006, Meier et al. 2008, Sihvonen et al. 2014, Jiang et al. 2014).

In the present study an overview of the Chinese Cyclidiinae is given with diagnostic characters for each genus and species, one new subspecies is described, two new synonyms are established, and one misidentification in Chu and Wang (1987) is revised. Also photos of external features and genitalia are provided of all Chinese species discussed. In addition, we discuss the application of the results of DNA barcoding for delimitation of five taxa of *Cyclidia*. As a result of this study five species and five subspecies of *Cyclidia*, and two species of *Mimozethes* are regarded as valid for the fauna of China.

## Materials and methods

*Morphology*. Studied specimens mainly belong to the Institute of Zoology, Chinese Academy of Sciences, Beijing, China (IZCAS) and the Natural History Museum, London, United Kingdom BMNH. Terminology for wing venation follows the Comstock-Needham System (Comstock 1918), and that of the genitalia is based on Klots (1970), Nichols (1989) and Kristensen (2003). Photographs of the moths were taken with digital cameras. Composite sharp images were generated using Auto-Montage software version 5.03.0061 (Synoptics Ltd). The plates were compiled using Adobe Photoshop software.

*DNA-Barcoding*. Prior to DNA sequencing, one or two legs were removed from several specimens of each of five examined taxa (*Cyclidia substigmaria substigmaria*, *Cyclidia rectificata rectificata*, *Cyclidia fractifasciata fractifasciata*, *Cyclidia fractifasciata indistincta*, *Cyclidia orciferaria*). DNA extraction was done using Qiagen DNeasy Blood and Tissue Kit (Qiagen, Beijing, China). The primers for the amplification of the 658 bp fragment were LepF1 (5’-ATTCAACCAATCATAAAGATATTGG-3’), LepR1 (5’-TAAACTTCTGGATGTCCAAAAAATCA-3’) (Hebert et al. 2004a). The PCR reactions were performed using the standard procedure described by Hebert et al. (2004a). The PCR products were detected by 1% agarose gel electrophoresis and directly sequenced with ABI PRISM 3730xl capillary sequencers. The amplification and sequencing for some dried material (Sequence ID begins with “DB”) were carried out in BGI-Shenzhen, (China) using standard protocols described in Hebert et al. (2004a). Forward and reverse nucleotide sequences were assembled in SeqMan 5.01 (DNASTAR, Inc. 1996). The assembled sequences were aligned and manually edited in MEGA 5.0 (Tamura et al. 2011). The neighbor-joining (NJ) tree (Saitou and Nei 1987) was reconstructed based on Kimura 2-parameter (K2P) distances (Kimura 1980) using MEGA 5.0. All the sequences have been deposited in GenBank under accession numbers, and their full data including images and are in the Barcode of Life Database (http://www.boldsystems.org; see Ratnasingham and Hebert 2007) (Table 1).

**Table 1.** *Cyclidia* species included in this study with GenBank accession numbers and BOLD process ID.

Taxa	Sequence ID	Collecting locality	Collecting date	GenBank accession no.	BOLD process ID
*Cyclidia substigmaria substigmaria*	DB00162	West Tianmushan, Zhejiang	Jul. 2003	KR872896	CLDC001-15
	DB00173 DB00174	Wuzhishan, Hainan Lingshui, Hainan	May 2007 May 2007	KR872897 KR872898	CLDC002-15 CLDC003-15
	DB00181	Baotianman, Henan	Aug. 2008	KR872899	CLDC004-15
	DB00182	Luoyang, Henan	Aug. 2006	KR872900	CLDC005-15
	DB00184	Baoshan, Yunnan	Aug. 2007	KR872901	CLDC006-15
	DB00189	Yanling, Hunan	Jul. 2008	KR872902	CLDC007-15
	IOZ LEP M 01129	Mengla, Yunnan	Jul. 2013	KR872903	CLDC008-15
	IOZ LEP M 01134	Tengchong, Yunnan	Aug. 2013	KR872904	CLDC009-15
	IOZ LEP M 01304	West Tianmushan, Zhejiang	Jul. 2011	KR872905	CLDC010-15
	IOZ LEP M 08961	Mengla, Yunnan	Jul. 2013	KR872906	CLDC011-15
	IOZ LEP M 09195	Qushi, Yunnan	Aug. 2013	KR872907	CLDC012-15
	IOZ LEP M 16605	Kangxian, Gansu	Aug. 2014	KR872908	CLDC013-15
	IOZ LEP M 16606	Kangxian, Gansu	Aug. 2014	KR872909	CLDC014-15
	IOZ LEP M 16607	Kangxian, Gansu	Aug. 2014	KR872910	CLDC015-15
	IOZ LEP M 16608	Kangxian, Gansu	Aug. 2014	KR872911	CLDC016-15
	IOZ LEP M 17993	Liuku, Yunnan	Sep. 2014	KR872912	CLDC017-15
	IOZ LEP M 17994	Liuku, Yunnan	Sep. 2014	KR872913	CLDC018-15
	IOZ LEP M 02790	Guilin, Guangxi	Apr. 1952	KR872914	CLDC019-15
*Cyclidia rectificata rectificata*	DB00226	Bomi, Tibet	Aug. 2005	KR872923	CLDC020-15
	DB00228	Mêdog, Tibet	Aug. 2006	KR872924	CLDC021-15
	DB00229	Mainling, Tibet	Aug. 2006	KR872925	CLDC022-15
	IOZ LEP M 03475	Zayü, Tibet	Aug. 2014	KR872926	CLDC023-15
	IOZ LEP M 03476	Zayü, Tibet	Aug. 2014	KR872927	CLDC024-15
	IOZ LEP M 03477	Zayü, Tibet	Aug. 2014	KR872928	CLDC025-15
	IOZ LEP M 16015	Zayü, Tibet	Aug. 2014	KR872929	CLDC026-15
*Cyclidia fractifasciata fractifasciata*	IOZ LEP M 00657	Pianma, Yunnan	May 2011	KR872930	CLDC027-15
	IOZ LEP M 00683	Pianma, Yunnan	May 2011	KR872931	CLDC028-15
	IOZ LEP M 07012	Pianma, Yunnan	May 2011	KR872932	CLDC029-15
	IOZ LEP M 07013	Pianma, Yunnan	May 2011	KR872933	CLDC030-15
*Cyclidia fractifasciata indistincta*	IOZ LEP M 16601	Kangxian, Gansu	Aug. 2014	KR872934	CLDC031-15
	IOZ LEP M 16602	Kangxian, Gansu	Aug. 2014	KR872935	CLDC032-15
	IOZ LEP M 16603	Kangxian, Gansu	Aug. 2014	KR872936	CLDC033-15
	IOZ LEP M 16604	Kangxian, Gansu	Aug. 2014	KR872937	CLDC034-15
	IOZ LEP M 09387	Wushan, Chongqing	Jul. 2013	KT250118	CLDC035-15
*Cyclidia orciferaria*	DB00202	Bawangling, Hainan	May 2007	KR872915	CLDC036-15
	DB00203	Wuzhishan, Hainan	Apr. 2008	KR872916	CLDC037-15
	DB00210	Yanling, Hunan	Jul. 2008	KR872917	CLDC038-15
	DB00211	Yanling, Hunan	Jul. 2008	KR872918	CLDC039-15
	DB00213	Shixing, Guangdong	Jun. 2008	KR872919	CLDC040-15
	DB00216	Baoshan, Yunnan	Aug. 2007	KR872920	CLDC041-15
	IOZ LEP M 01208	West Tianmushan, Zhejiang	Jul. 2011	KR872921	CLDC042-15
	IOZ LEP M 01324	West Tianmushan, Zhejiang	Jul. 2011	KR872922	CLDC043-15

## Results

### Taxonomy
Cyclidiinae Warren, 1922
Cyclidiinae Warren, 1922: 444.

#### Cyclidia

Taxon classificationAnimaliaLepidopteraDrepanidae

Guenée, 1858

Cyclidia Guenée, 1858: 62. Type species: *Cyclidia substigmaria* (Hübner, 1831), by monotypy.
Nelcynda Walker, 1862a: 1142. Type species: *Nelcynda rectificata* Walker, 1862, by monotypy.
Ciclidia Chou & Xiang, 1984: 159. [Incorrect spelling of *Cyclidia* Guenée.]

##### Generic characters.

**Figures 1–3.**
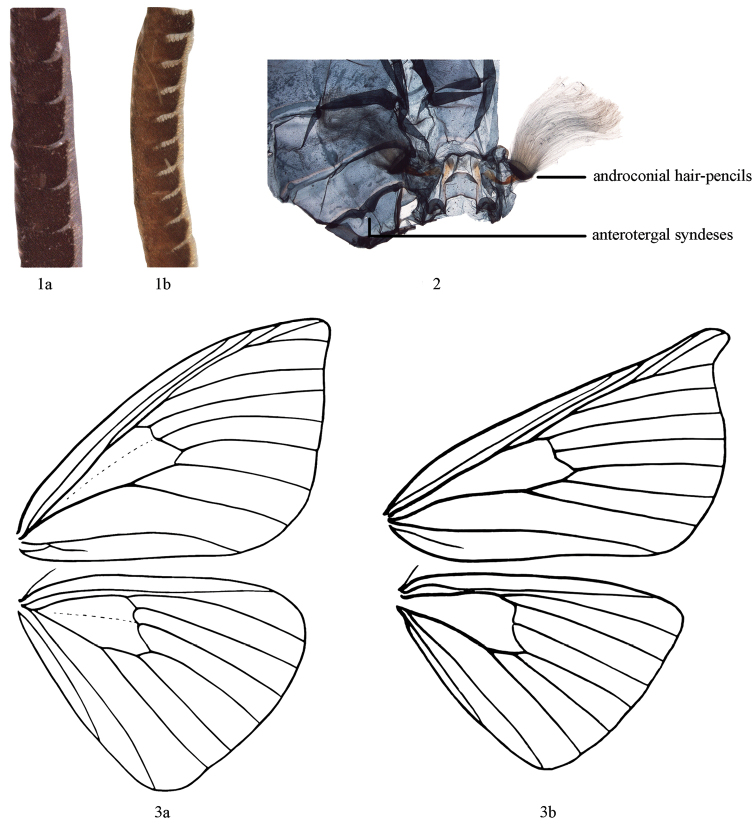
**1** Male antennae **a** *Cyclidia substigmaria* **b** *Mimozethes angula* **2** Anterotergal syndeses and androconial hair-pencils **3** Wing venation (from Chu and Wang, 1991) **a** *Cyclidia substigmaria* **b** *Mimozethes angula*.

***Head*.** Antennae lamellate, partly unipectinate, rami very short (Fig. 1a). Frons not protruding. Labial palpi with third segment distinct, up-curved. ***Thorax*.** Hind tibia with two pairs of spurs. Apex of forewing often rounded, sometimes pointed and protruding. Wing colour usually white or grey (except *Cyclidia orciferaria*); antemedial and postmedial lines of forewing double; medial line of forewing broad; terminal lines of both wings usually double, sometimes single (e.g. *Cyclidia substigmaria*, *Cyclidia rectificata*, and *Cyclidia diehli* Lutz & Kobes, 2002). Venation (Fig. 3a). Forewing with R_1_ separate, R_2–4_ and R_5_ stalked, R_2_ and R_3+4_ stalked, R_5_ and M_1_ separate, M_2_ arising from middle of discocellulars; Hind wing with Sc+R_1_ close to Rs beyond distal cell, then far from Rs, M_2_ arising from middle of discocellulars. Anterotergal syndeses developed at anterior margin of 2^nd^ tergum (Fig. 2). A pair of androconial hair-pencils present on 2^nd^ sternum of male (Fig. 2). ***Male genitalia*.** Uncus triangular; socii developed, often sclerotized (except *Cyclidia orciferaria*), sometimes with small setose process at base (e.g. *Cyclidia pitimani* (Moore, 1886), *Cyclidia sericea* and *Cyclidia diehli*); gnathos connected at middle and with median process narrow and triangular; valva simple and broad; juxta deeply concaved posteriorly; saccus short and broad, rounded terminally; phallus slightly curved; vesica without cornuti. ***Female genitalia*.** Papillae anales broad and rounded; lamella postvaginalis usually well developed; ductus bursae very long and narrow, with a colliculum; corpus bursae oval, with a paired band-like spinose signa.

##### Diagnosis.

*Cyclidia* is quite different from *Mimozethes* externally and in the genitalia. For example, externally, the rami of the antennae are much shorter; the species of *Cyclidia* are much larger, and the postmedial lines of forewing are often double, while in *Mimozethes*, it is single and forms a “>” shaped protrusion near R_5_; in the male genitalia, the socii are well developed in *Cyclidia*, but absent in *Mimozethes*; the sacculus unmodified in *Cyclidia* but forming a process in *Mimozethes*; in the female genitalia, the signa are a paired band-like sclerotization in *Cyclidia*, but absent in *Mimozethes*.

##### Distribution.

China, Japan, Korean Peninsula, south and southeast Asia.

##### Key to Chinese *Cyclidia* species

**Table:** 

1	Wings colour white or grey	**2**
–	Wings colour blackish brown	***Cyclidia orciferaria***, Figs 18–19
2	Discal spots on hind wing distinct	**3**
–	Discal spots on hind wing indistinct	**4**
3	Discal spots on hind wing dark grey	***Cyclidia substigmaria substigmaria***, Figs 4–8
–	Discal spots on hind wing black	***Cyclidia substigmaria intermedia***, Fig. 9
4	Terminal lines of both wings single	***Cyclidia rectificata rectificata***, Figs 10–11
–	Terminal lines of both wings double	**5**
5	Outer margin of forewing medial line forming an right angle below M_3_	**6**
–	Outer margin of forewing medial line not forming an right angle below M_3_	***Cyclidia pitimani***, Figs 12–13
6	Outer line of antemedial line and inner line of postmedial line of forewing distinct	***Cyclidia fractifasciata fractifasciata***, Figs 14–15
–	Outer line of antemedial line and inner line of postmedial line of forewing invisible	***Cyclidia fractifasciata indistincta***, Figs 16–17

**Figures 4–11.**
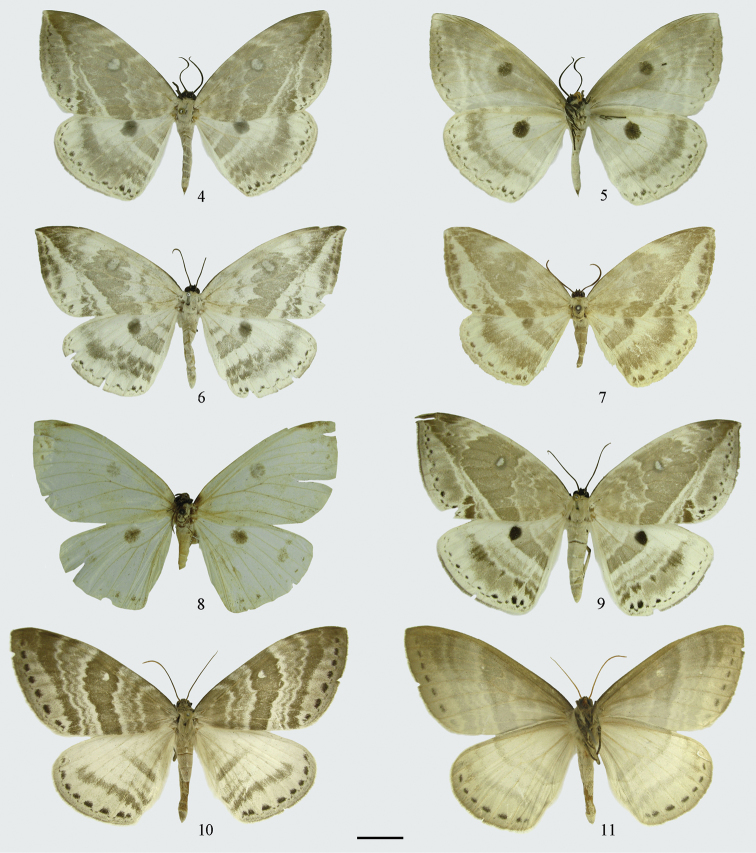
Adults. **4–9** *Cyclidia substigmaria substigmaria* **4** male (with dot-like and wavy submarginal line of the forewing, Yunnan) **5** ditto, underside **6** male (with faint, broad and interrupted submarginal line of the forewing, Zhejiang) **7** male (holotype of *Cyclidia substigmaria brunna*, Sichuan) **8** male (holotype of *Cyclidia tetraspota*, Yunnan) **9** *Cyclidia substigmaria intermedia*, male (Tibet) **10–11** *Cyclidia rectificata* **10** male (Tibet) **11** ditto, underside. Scale bar: 1 cm.

**Figures 12–23.**
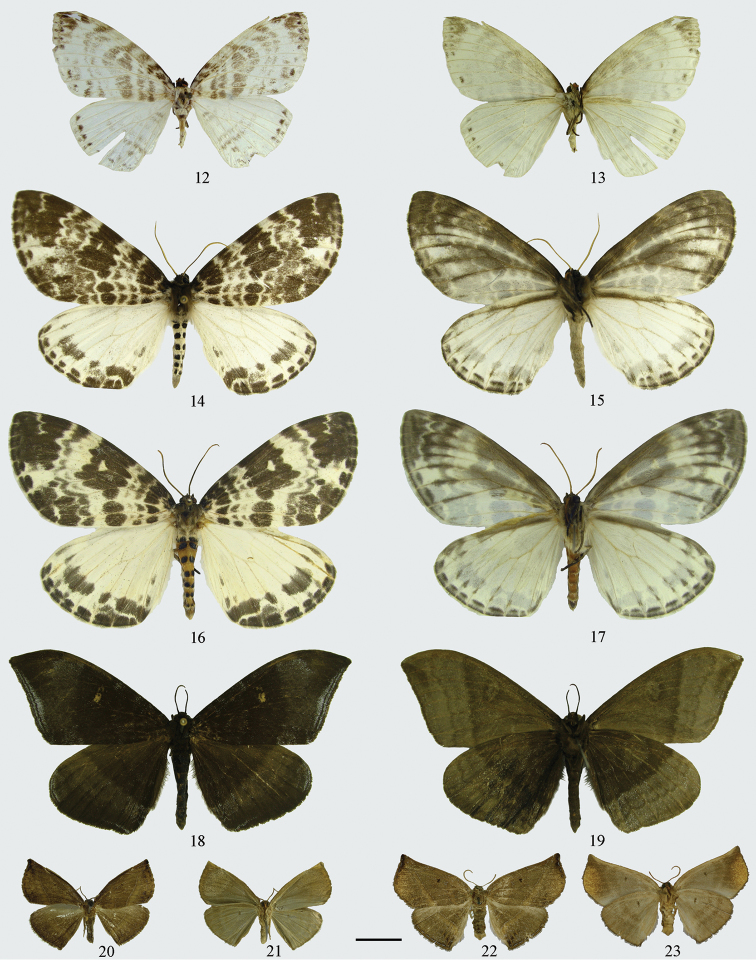
Adults. **12–13** *Cyclidia pitimani* **12** male (Yunnan) **13** ditto, underside **14–15** *Cyclidia fractifasciata fractifasciata* **14** male (Yunnan) **15** ditto, underside **16–17** *Cyclidia fractifasciata indistincta* subsp. n. **16** male (holotype, Gansu) **17** ditto, underside **18–19** *Cyclidia orciferaria* **18** male (Hainan) **19** ditto, underside **20–21** *Mimozethes angula* **20** male (holotype, Sichuan) **21** ditto, underside **22–23** *Mimozethes lilacinaria* **22** male (holotype, Sichuan) **23** ditto, underside. Scale bar: 1 cm.

#### Cyclidia substigmaria

Taxon classificationAnimaliaLepidopteraDrepanidae

(Hübner, 1831)

Euchera substigmaria Hübner 1831: 29. pl. 90, figs 519, 520. Syntypes, China.
Cyclidia substigmaria : Guenée 1858: 63.
Abraxas capitata Walker, 1862a: 1121. Holotype ♀, China: Hong Kong (BMNH).
Euchera capitata : Strand 1911: 196.
Cyclidia substigmaria brunna Chu & Wang, 1987: 205. Holotype ♂, China: Sichuan: Emeishan, Qingyinge (IZCAS). **Syn. n.**
Cyclidia tetraspota Chu & Wang, 1987: 206. Holotype ♂, China: Yunnan: Xishuangbanna, Yunjinghong (IZCAS). **Syn. n.**

##### Diagnosis.

In external appearance, this species is distinguishable from other congeners by the following characters: the discal spots of hind wing are very distinct on the upper side and the underside; the discal spot of the forewing is covered with white scales on the upper side; two greyish brown markings are present inside the anal angle of the forewing. The male genitalia of the species are close to those of *Cyclidia rectificata*, but the terminal part of the uncus and the socii are narrower; the vesica is much more scobinate. In the female genitalia, the two signa are close to each other posteriorly, while in *Cyclidia rectificata*, they are almost parallel.

##### Remarks.

There are five subspecies of *Cyclidia substigmaria*:

*Cyclidia substigmaria substigmaria* (Hübner, 1831), most parts of China and Vietnam;

*Cyclidia substigmaria intermedia* Prout, 1918 in Tibet;

*Cyclidia substigmaria nigralbara* Warren, 1914 in Japan and Korean Peninsula;

*Cyclidia substigmaria modesta* Bryk, 1943 in Myanmar;

*Cyclidia substigmaria superstigmaria* Prout, 1918 in India and Nepal.

##### Distribution.

China, Japan, Korean Peninsula, India, Nepal, Myanmar, Vietnam.

##### Biological notes.

Sugi (1987) and Holloway (1998) mentioned that larval Cyclidiinae may be uniquely associated with the plant family Alangiaceae (now incorporated in Cornaceae). However, *Cyclidia substigmaria* also has been recorded from Malvaceae (*Hibiscus cannabinus* L.) (Chu 1981, Chu and Wang 1987, 1991, Kadoorie Farm and Botanical Garden 2004). The morphology of the eggs, larva, pupa and life history of *Cyclidia substigmaria* were described in detail by Zhou and Wang (1985), Chu and Wang (1991) and Yan et al. (2009).

#### Cyclidia substigmaria substigmaria

Taxon classificationAnimaliaLepidopteraDrepanidae

(Hübner, 1831)

Figs 4–8 , 24–28 , 37–39 , 48–50

##### Diagnosis.

The subspecies is very similar to *Cyclidia substigmaria intermedia*, but differs externally by the paler discal spot of the hind wing and the two less distinct markings inside the anal angle of the forewing.

##### Type material examined.

**CHINA: Sichuan** (IZCAS): 1♂ (Holotype of *Cyclidia substigmaria brunna*), Emeishan, Qingyinge, 800–1000 m, 17.V.1957, coll. Wang Zongyuan. **Zhejiang** (IZCAS): 1♀ (Allotype of *Cyclidia substigmaria brunna*), Hangzhou, 4.V.1975, coll. Zhang Baolin. **Fujian** (IZCAS): 3♂ (Paratypes of *Cyclidia substigmaria brunna*), Wuyishan, 6–21.V.1983, coll. Wang Linyao. **Yunnan** (IZCAS): 1♀ (Paratypes of *Cyclidia substigmaria brunna*), Liuku, 2500 m, 23.V.1981, coll. Liao Subai; 1♂ (Holotype of *Cyclidia tetraspota*), Xishuangbanna, Yunjinghong, 650 m, 22.VI.1959, coll. Meng Xuwu; 1♀ (Allotype of *Cyclidia tetraspota*), Yiwubanna, Menglun, 650 m, 23.VII.1959, coll. Zhang Facai; 1♂ (Paratype of *Cyclidia tetraspota*), ibidem, 28.V.1958, coll. Wang Shuyong. **Hainan** (IZCAS): 1♂ (Paratype of *Cyclidia tetraspota*), Wanning, 10 m, 9.IV.1960, coll. Li Zhenfu. **Guangxi** (IZCAS): 1♂ (Paratype of *Cyclidia tetraspota*), Guilin, Liangfeng, 20.IV.1952. **Hongkong** (BMNH): 1 ♀, collector and collecting date unknown (Holotype of *Cyclidia substigmaria capitata*).

##### Additional material examined.

**CHINA: Henan** (IZCAS): 1♂, Luoyang, Huaguoshan, 4.VIII.2006, coll. Song Hao; 1♀, Baiyunshan, 1400 m, 27.VII.2003, coll. Lu Yanan; 1♂, Jigongshan, 25.VI.1984. **Shaanxi** (IZCAS): 2♂1♀, Ningshan, Guanghuojie, 1189 m, 28.VII.2014, coll. Liu Shuxian and Ban Xiaoshuang; 1♂, Zhashui, Yingpanzhen, 980 m, 31.VII.2014, Liu Shuxian and Ban Xiaoshuang; 1♂, Xunyang, Bailiuzhen, 386 m, 3.VIII.2014, coll. Liu Shuxian and Ban Xiaoshuang. **Gansu** (IZCAS): 1♂, Wenxian, Qiujiaba, 2200–2350 m, 29.VI.1998, coll. Yuan Decheng; 1♀, Kangxian, Baiyunshan, 1250–1750 m, 12.VII.1998, coll. Wang Shuyong; 1♂7♀, Kangxian, Yangba, Meiyuangou, 1000 m, 13.VIII.2014, coll. Xue Dayong & Ban Xiaoshuang; 1♀, Wenxian, Lukou, 22.V.1987. **Jiangsu** (IZCAS): 7♂4♀, Chemo, 22.IV–2.V.1935, coll. O. Piel. **Anhui** (IZCAS): 1♀, Linzongchang, IX.1970, coll. Mai Weiqiang; 2♀, Yuexi, Linyeju, 11.IX.1982, coll. Zhou Tiying. **Zhejiang** (IZCAS): 5♂3♀, Lin’an, West Tianmushan, 400–1506 m, 6.IX.1981, 26–30.VII.2003, 27.VII.2011, coll. Xue Dayong et al.; 15♂1♀, Tianmushan, 15–25.VI.1936, 25–30.VIII.1947, 22.VIII.1972, 28–31.VII.1998, coll. O. Piel et al.; 1♂1♀, Hangzhou, 4.V.1975, 1981, coll. Zhang Baolin; 1♂, Qingyuan, Fengyangshan, Datianping, 1290 m, 6–10.VIII.2003, coll. Han Hongxiang. **Hubei** (IZCAS): 1♂, Shennongjia, Muyu, 22.VII.1998, coll. Zhou Hongzhang; 1♀, Shennongjia, Dalongtan, 2700 m, 27.VII.1998, coll. Zhou Haisheng; 1♂, Xingshan, Longmenhe, 1300 m, 12.IX.1994, coll. Song Shimei; 4♀, Xuan’en, 650 m, 25.V.1989, coll. Li Wei; 1♀, Hefeng, Fenshuiling Linchang, 31.VII.1989, coll. Li Wei. **Jiangxi** (IZCAS): 1♀, Yifeng, Yuanqian, 8.IX.1959. **Hunan** (IZCAS): 1♀, Yanling, Taoyuandong, 631 m, 4–8.VII.2008, coll. Chen Fuqiang; 1♀, Fenghuang, 15.IX.1988, coll. Song Shimei; 1♀, Cili, 3.IX.1988, coll. Song Shimei. **Fujian** (IZCAS): 11♂9♀, Wuyishan, 26.IV–14.VI.1983, coll. Wang Linyao and Zhang Baolin; 1♂, Xinkou, 15.VI.1981, coll. Lin Yibiao; 2♂1♀, Jianyang, Huangkeng, 270–950 m, 23.IV–1.V.1960, coll. Jiang Shengqiao and Zuo Yong; 1♀, Chong’an, Xingcun, Guadun, 840–1210 m, 25.VIII.1960, coll. Ma Chenglin; 1♀, Chong’an, Xingcun, Sangang, 740 m, 17.V.1960, coll. Zhang Yiran. **Guangdong** (IZCAS): 1♂, Guangzhou, 8.VI.1973, coll. Zhang Baolin; 4♂5♀, Guangzhou, Sanyuanli, 27.IV.1958, coll. Wang Linyao. **Hainan** (IZCAS): Wanning, 10 m, 14.IV.1960, coll. Li Changqing; 3♂, Xinglong, 24.III.1963, IV.1963, coll. Zhang Baolin; 3♂, Lingshui, Diaoluoshan, 4–5.V.2007, coll. Han Hongxiang; 1♀, Wuzhishan, Shuiman, 600 m, 12.V.2007, coll. Han Hongxiang; 1♀, Baisha, Yinggeling, 434 m, 3–4.XII.2007, coll. Li Jing; 1♀, Jianfengling, Tianchi, 3.III.1982, coll. Long Yongcheng. **Guangxi** (IZCAS): 1♂1♀, Jinxiu, Luoxiang, 200–400 m, 1–16.V.1999, coll. Huang Fusheng and Han Hongxiang; 1♀, Jinxiu, Yonghe, 500 m, 12.IV.1999, coll. Han Hongxiang; 1♀, Jinxiu, Jinzhong Gonglu, 1100 m, 12.V.1999, coll. Li Wenzhu; 2♂, Guilin, Yanshan, 26.IX.1958, 19.XI.1959; 1♂5♀, Fangcheng, Fulong, 240–260 m, 1.III.1998, 19–20.IV.1998, coll. Li Wenzhu and Wu Chunsheng; 1♂, Napo, Nianjing, 900 m, 11.IV.1998, coll. Wu Chunsheng; 1♀, Napo, Defu, 1350 m, 19.VI.2000, coll. Yao Jian; 1♀, Napo, Nonghua, 990 m, 13.IV.1998, coll. Li Wenzhu; 1♀, Napo, Baihe, 540 m, 8.IV.1998, coll. Qiao Gexia; 1♂, Pingxiang, 230 m, 8.VI.1976, coll. Zhang Baolin; 2♀, Longsheng, 10–11.VI.1980, coll. Zhong Tiesen and Song Shimei; 2♀, Daxin, Xialei, 680 m, 31.III.1998, coll. Li Wenzhu; 2♂, Longzhou, Nonggang, 195 m, 15–17.VII.2013, coll. Liu Shuxian and Li Xinxin. **Sichuan** (IZCAS): 1♀, Emeishan, Baoguosi, 550–750 m, 8.IV.1957, coll. Wang Zongyuan; 1♂, Emeishan, 580–1100 m, 22.VI.1955, coll. Zi Yunzhen; 36♂34♀, Emeishan, Qingyinge, 800–1000 m, 17.IV–20.V.1957, 19.IX–28.X.1957, coll. Zhu Fuxing et al.; 1♀, Yanyuan, Jinhe, 2.VII.1984, coll. Chen Yixin. **Guizhou** (IZCAS): 1♀, Sinan, 350 m, 9.V.1983, coll. Liu Yanxian; 1♂, Koei-Yang, 5.IX.1935. **Yunnan** (IZCAS): 2♂7♀, Xishuangbanna, Mengna, 550 m, 22–30.VI.1959, coll. Zhang Yiran and Li Zhenfu; 1♂3♀, Xiaomengyang, 850–1000 m, 6.V.1957, 12.VII–22.VIII.1957, 10.X.1957, coll. Wang Shuyong et al.; 1♂1♀, Xishuangbanna, Menghun, 160–750 m, 4.VI.1958, coll. Meng Xuwu et al.; 1♀, Xishuangbanna, Yunjinghong, 650 m, 3.VII.1957, coll. Wang Shuyong; 2♂6♀, Xishuangbanna, Mengla, 620–650 m, 2.V–6.VI.1959, coll. Zhang Yiran et al.; 6♂9♀, Mengla, Menglun, 650–665 m, 22–29.X.1958, 3.IV–18.V.1964, 29.VII.2013, coll. Wang Shuyong et al.; 1♂, Xishuangbanna, Menghai, 1200–1600 m, 18.VII.1958, coll. Wang Shuyong; 2♀, Xishuangbanna, Ganlanba, 560 m, 9–10.VII.1958, coll. Li Chuanlong; 1♂, Xishuangbanna, Bubang, 700 m, 14.IX.1993, coll. Yang Longlong; 1♀, Xishuangbanna, Yiwu, 800–1300 m, 13.VII.1959, coll. Pu Fuji; 6♂1♀, Baoshan, Baihualing, 1520 m, V.11–13.VIII.2007, coll. Wu Chunguang and Lang Songyun; 2♂1♀, Baoshan, Bawan, 1040–1100 m, 19–23.1992, 8–10.VIII.2007, 8–10.VIII.2013, coll. Wu Chunguang et al.; 2♂3♀, Baoshan, Xinujiang Hegu, 800–1000 m, 10–11.V.1955, coll. Xue Yufeng; 1♂, Tengchong, Qushi, Dabacun, 1873 m, 4.VIII.2013, coll. Liu Shuxian and Li Xinxin; 7♂1♀, Tengchong, Zhengding, 1833 m, 6–7.VIII.2013, coll. Liu Shuxian and Li Xinxin; 2♀, Tengchong, Heinitang, 1824 m, 26–27.VI.2014, coll. Li Xinxin and Pan Xiaodan; 1♀, Cheli, 620 m, 18.IV.1957, coll. Zang Lingchao; 2♂, Yuanyang, Nansha, 1100 m, 26.V.1979, coll. Luo Kezhong; 1♂1♀, Lushui, Liuku, 860–1220 m, 18–19.IX.2014, coll. Liang Hongbin; 2♂4♀, Lushui, Pianma, 1750–1980 m, 7.V.1981, 8–12.V.2011, 3–4.VII.2014, coll. Zhang Xuezhong et al.; 1♀, Jinping, Mengla, 500 m, 2.V.1956, coll. Huang Keren; 1♀, Jinping, Chang Potou, 1200 m, 23.V.1956, coll. Huang Keren. **Vietnam** (IZCAS): 1♀, Tonkin, Hoa-Binh, leg. A. de Cooman.

##### Variation.

The submarginal line of the forewing varies from dot-like and wavy to faint, broad and interrupted between veins. In the male genitalia, the terminal half of the costa vary from smooth (Fig. 27, IOZ LEP M 01129) to strongly protruding (Fig. 28, IOZ LEP M 08961) among the material on the same region.

**Figures 24–29.**
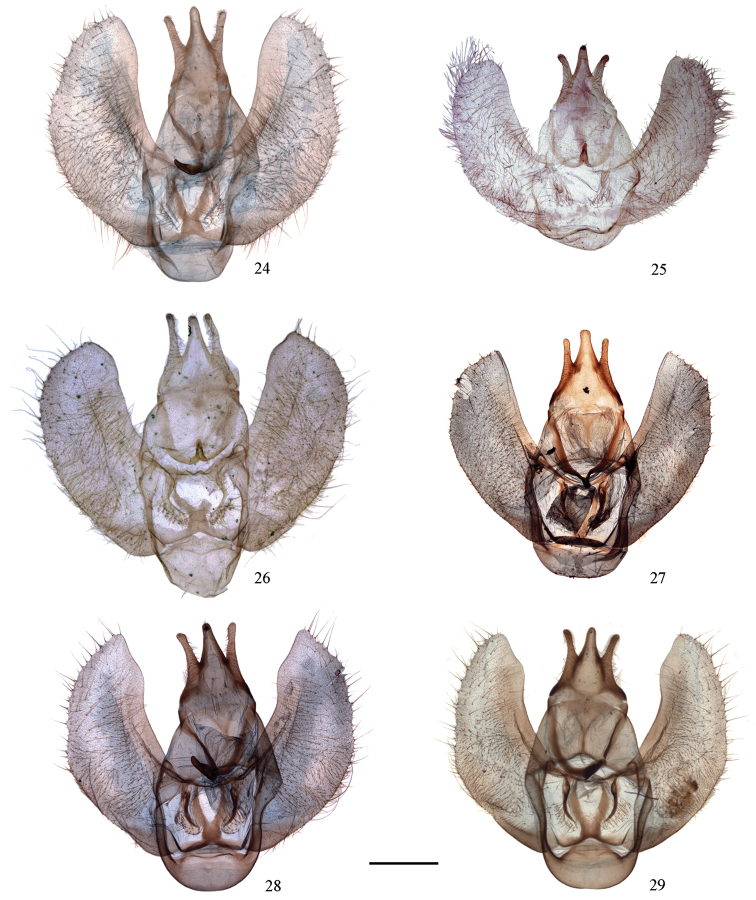
Male genitalia of *Cyclidia*. **24** *Cyclidia substigmaria substigmaria* (Baoshan, Yunnan, slide no. 41) **25** ditto (holotype of *Cyclidia substigmaria brunna*, Emeishan, Sichuan, slide no. 12) **26** ditto (holotype of *Cyclidia tetraspota*, Xishuangbanna, Yunnan, slide no. 10) **27** ditto (Xishuangbanna, Yunnan, slide no. 681) **28** ditto (Xishuangbanna, Yunnan, slide no. 683) **29** *Cyclidia substigmaria intermedia* (Tibet, slide no. 311). Scale bar: 1 mm.

##### Genetic data.

The distance to the nearest neighbour *Cyclidia rectificata* is 8.92%. The intrasubspecific divergence of the barcode region of *Cyclidia substigmaria substigmaria* ranges from 0%–2.6% (average distance 1%) (n = 19). Some specimens from Yunnan cluster together at some distance from all other specimens (Fig. 58). Despite the high divergence, no morphological characters were found which separate these populations.

##### Remarks.

After examining the types of *Cyclidia substigmaria brunna*, *Cyclidia tetraspota* and a long series of material collected near their type localities, it was found that the external and genital features of *Cyclidia substigmaria brunna* and *Cyclidia tetraspota* are nearly identical to those of *Cyclidia substigmaria substigmaria*. Barcodes of one paratype of *Cyclidia tetraspota* (IOZ LEP M 02790) and two specimens from type locality of *Cyclidia substigmaria brunna* (IOZ LEP M 17993 and 17994) were clustered within *Cyclidia substigmaria substigmaria* in the Neighbour Joining (NJ) tree with the genetic distances from 0.015%–2.6% (see fig. 58). Thus, *Cyclidia tetraspota* and *Cyclidia substigmaria brunna* are considered as junior synonyms of *Cyclidia substigmaria substigmaria*.

##### Distribution.

China (Henan, Shaanxi, Gansu, Jiangsu, Anhui, Zhejiang, Hubei, Jiangxi, Hunan, Fujian, Taiwan, Guangdong, Hainan, Hong Kong, Guangxi, Sichuan, Guizhou, Yunnan), Vietnam.

#### Cyclidia substigmaria intermedia

Taxon classificationAnimaliaLepidopteraDrepanidae

Prout, 1918

Figs 9 , 29 , 40 , 51

Cyclidia substigmaria intermedia Prout, 1918: 416. Holotype ♂, China: Tibet (BMNH).

##### Diagnosis.

See under *Cyclidia substigmaria substigmaria*.

##### Type material examined.

**CHINA: Tibet** (BMNH): 1♂ (Holotype), Tibet, collector and collecting date unknown, ex. Joicey Collection.

##### Additional material examined.

**CHINA: Tibet** (IZCAS): 1♂, Mêdog, Yarang, 1091 m, 20–23.VIII.2006, coll. Lang Songyun; 1♀, Mêdog, Beibung, 850 m, 24.VI.1983, coll. Han Yinheng; 2♀, Mêdog, 2750 m, 22.VIII.1982, coll. Han Yinheng; 1♀, Zayü, Dongyan, 1600 m, 17.VII.1973.

##### Genetic data.

No genetic data available.

##### Distribution.

China (Tibet).

#### Cyclidia rectificata

Taxon classificationAnimaliaLepidopteraDrepanidae

(Walker, 1862)

Nelcynda rectificata Walker, 1862a: 1142. Holotype 1♂, India: Sikkim (BMNH).
Cyclidia muricolaria Walker, 1862b: 1483. Holotype 1♀, India: Darjeeling (BMNH).
Cyclidia patulata Walker, 1866: 1537. Holotype ♀, India: Darjeeling (BMNH).
Chorodna rectificata : Cotes and Swinhoe 1888: 475.
Enchera rectificata : Hampson 1893: 328.
Cyclidia rectificata : Warren 1922: 445.

##### Diagnosis.

The species is very similar to *Cyclidia diehli* Lutz & Kobes, 2002 (Sumatra) externally, but can be distinguished by the blackish brown and more distinct forewing submarginal line. The most distinct differences are in the male genitalia: the terminal part of the uncus is much narrower and longer; a rounded process with short setae is absent on the basal part of each socius, while *Cyclidia diehli* has this character; the terminal part of the valva is much broader than that of *Cyclidia diehli*. The male and female genitalia are also similar to those of *Cyclidia substigmaria*, the diagnosis can be seen under *Cyclidia substigmaria substigmaria*.

##### Remarks.

There are two subspecies of *Cyclidia rectificata*. *Cyclidia rectificata rectificata* (Walker, 1862) is distributed in China and India, and *Cyclidia rectificata malaisei* Bryk, 1943 is distributed in Myanmar.

##### Distribution.

China, India, Myanmar.

#### Cyclidia rectificata rectificata

Taxon classificationAnimaliaLepidopteraDrepanidae

(Walker, 1862)

**Figures 30–34.**
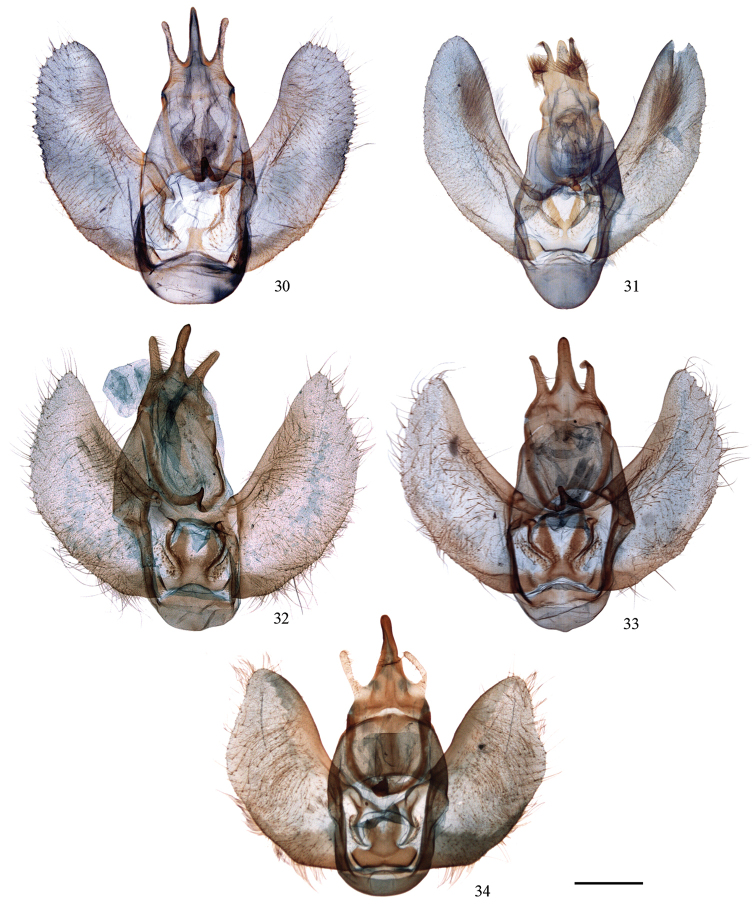
Male genitalia of *Cyclidia*. **30** *Cyclidia rectificata* (Tibet, slide no. 2) **31** *Cyclidia pitimani* (Yunnan, slide no. 9) **32** *Cyclidia fractifasciata fractifasciata* (Yunnan, slide no. 724) **33** *Cyclidia fractifasciata indistincta* subsp. n. (paratype, Gansu, slide no. 721) **34** *Cyclidia orciferaria* (Hainan, slide no. 728). Scale bar: 1 mm.

Figs 10 , 11 , 30 , 41 , 52

##### Diagnosis.

See under *Cyclidia rectificata*.

##### Material examined.

**CHINA: Yunnan** (IZCAS): 1♂, Tengchong, Heinitang, 1930 m, 28–30.V.1992, coll. Xue Dayong. **Tibet** (IZCAS): 7♀, Nyalam, Zham, 2250 m, 12–20.V.1974, coll. Zhang Xuezhong; 1♂, Cona, 2800 m, 8.VIII.1974, coll. Huang Fusheng; 1♂, Zham, 2200 m, 25.VI.1975, coll. Wang Ziqing; 1♀, Gyirong, 2800 m, 26.VIII.1975, coll. Wang Ziqing; 3♀, Bomi, Yi’ong, 2300 m, 23–29.VIII.1983, coll. Han Yinheng; 2♂5♀, Nyingchi, Bomi, Tangmai, 2100 m, 29–31.VIII.2005, coll. Wang Xuejian; 3♂1♀, Nyingchi, Pêlung, 2115 m, 1–2.IX.2005, coll. Wang Xuejian; 4♂3♀, Zayü, Shang Zayü, 1812–1960 m, 21–23.VIII.2005, 10–11.VIII.2014, coll. Wang Xuejian, Cheng Rui and Cui Le; 1♂1♀, Zayü, Rongcheng Binguan, 2178 m, 8–12.VIII.2014, coll. Cheng Rui and Cui Le; 2♀, Mainling, Pai, 2883 m, 4–6.VIII.2006, coll. Lang Songyun; 8♂11♀, Mêdog, Lage, 3213 m, 7–8.VIII.2006, coll. Lang Songyun; 3♂2♀, Mêdog, Dayandong, 2880 m, 9.VIII.2006, coll. Lang Songyun; 2♂, Mêdog, Hanmi, 2095 m, 10–11.VIII.2006, coll. Lang Songyun; 2♀, Mêdog, Pomo Gonglu 80K, 2118 m, 24–25.VIII.2006, coll. Lang Songyun.

**Figures 35–47.**
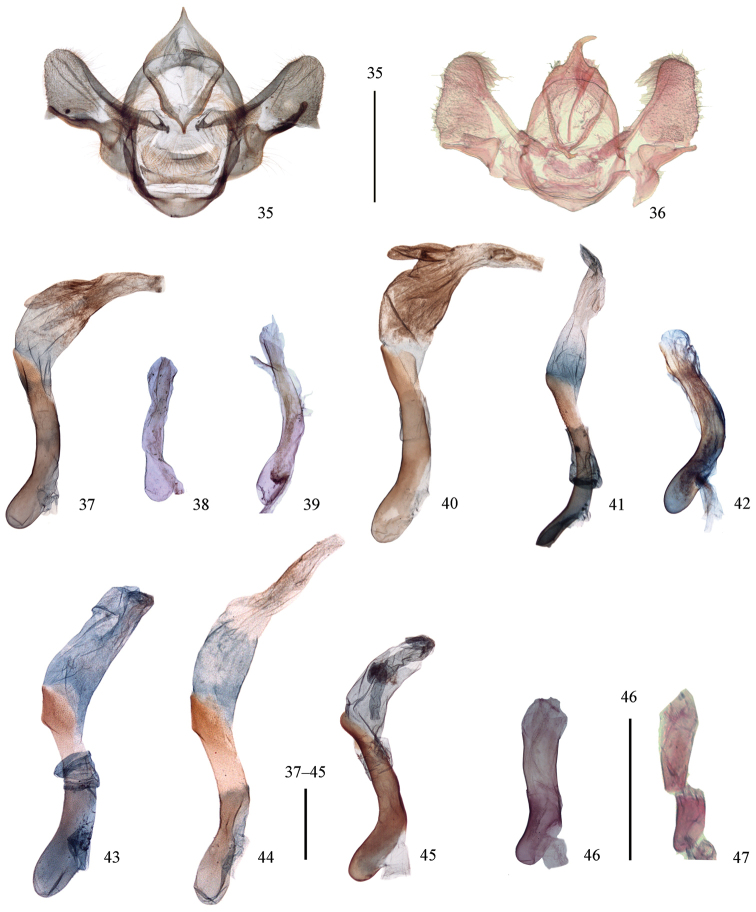
**35–36** Male genitalia of *Mimozethes*. **35** *Mimozethes angula* (paratype, Sichuan, slide no. 34) **36** *Mimozethes lilacinaria* (Sichuan, BMNH, slide No. 304) **37–47** Phallus **37** *Cyclidia substigmaria substigmaria* (Tengchong, Yunnan, slide no. 682) **38** ditto (holotype of *Cyclidia substigmaria brunna*, Emeishan, Sichuan, slide no. 12) **39** ditto (holotype of *Cyclidia tetraspota*, Xishuangbanna, Yunnan, slide no. 10) **40** *Cyclidia substigmaria intermedia* (Tibet, slide no. 311) **41** *Cyclidia rectificata* (Tibet, slide no. 727) **42** *Cyclidia pitimani* (Yunnan, slide no. 9) **43** *Cyclidia fractifasciata fractifasciata* (Yunnan, slide no. 724) **44** *Cyclidia fractifasciata indistincta* subsp. n. (paratype, Gansu, slide no. 721) **45** *Cyclidia orciferaria* (Hainan, slide no. 728) **46** *Mimozethes angula* (holotype, Sichuan, slide no. 19) **47** *Mimozethes lilacinaria* (Sichuan, BMNH, slide No. 304). Sscale bars: 1 mm.

**Figures 48–57.**
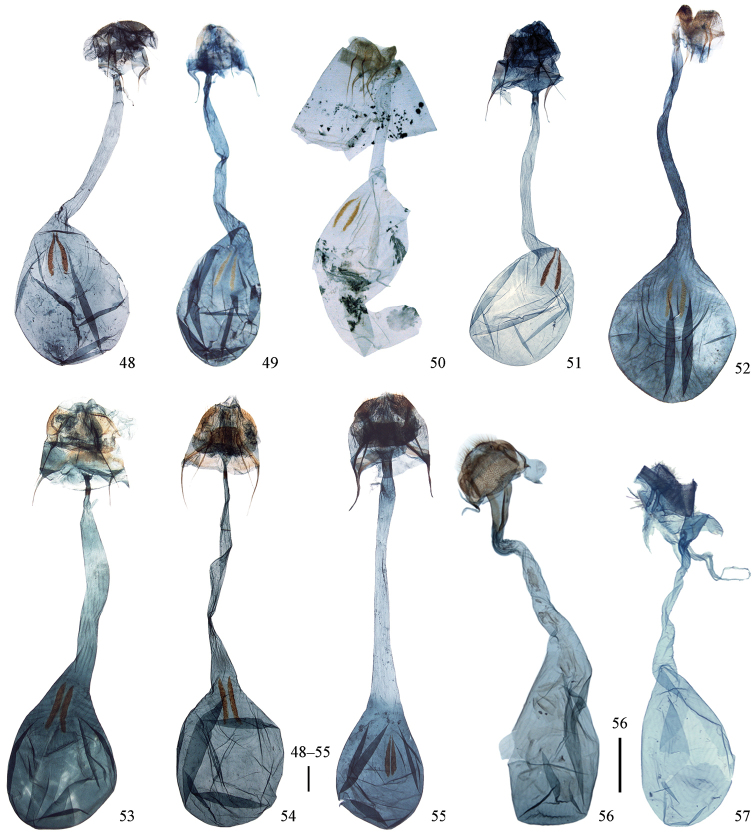
Female genitalia. **48** *Cyclidia substigmaria substigmaria* (Henan, sldie. no. 726) **49** ditto (Jiangsu, slide. no. 33) **50** ditto (paratype of *Cyclidia tetraspota*, Xishuangbanna, Yunnan, slide no. 36) **51** *Cyclidia substigmaria intermedia* (Tibet, slide no. 685) **52** *Cyclidia rectificata* (Tibet, slide no. 3) **53** *Cyclidia fractifasciata fractifasciata* (Yunnan, slide no. 725) **54** *Cyclidia fractifasciata indistincta* subsp. n. (paratype, Gansu, slide no. 722) **55** *Cyclidia orciferaria* (Hainan, slide no. 729) **56** *Mimozethes angula* (Henan, slide no. 288) **57** *Mimozethes lilacinaria* (Sichuan, slide no. 280). Sscale bars: 1 mm.

##### Genetic data.

The intraspecific divergence of the barcode region of *Cyclidia rectificata rectificata* is 0% (average distance 0%) (n = 7). The distance to the nearest neighbour *Cyclidia substigmaria* is 8.92%.

**Figure 58.**
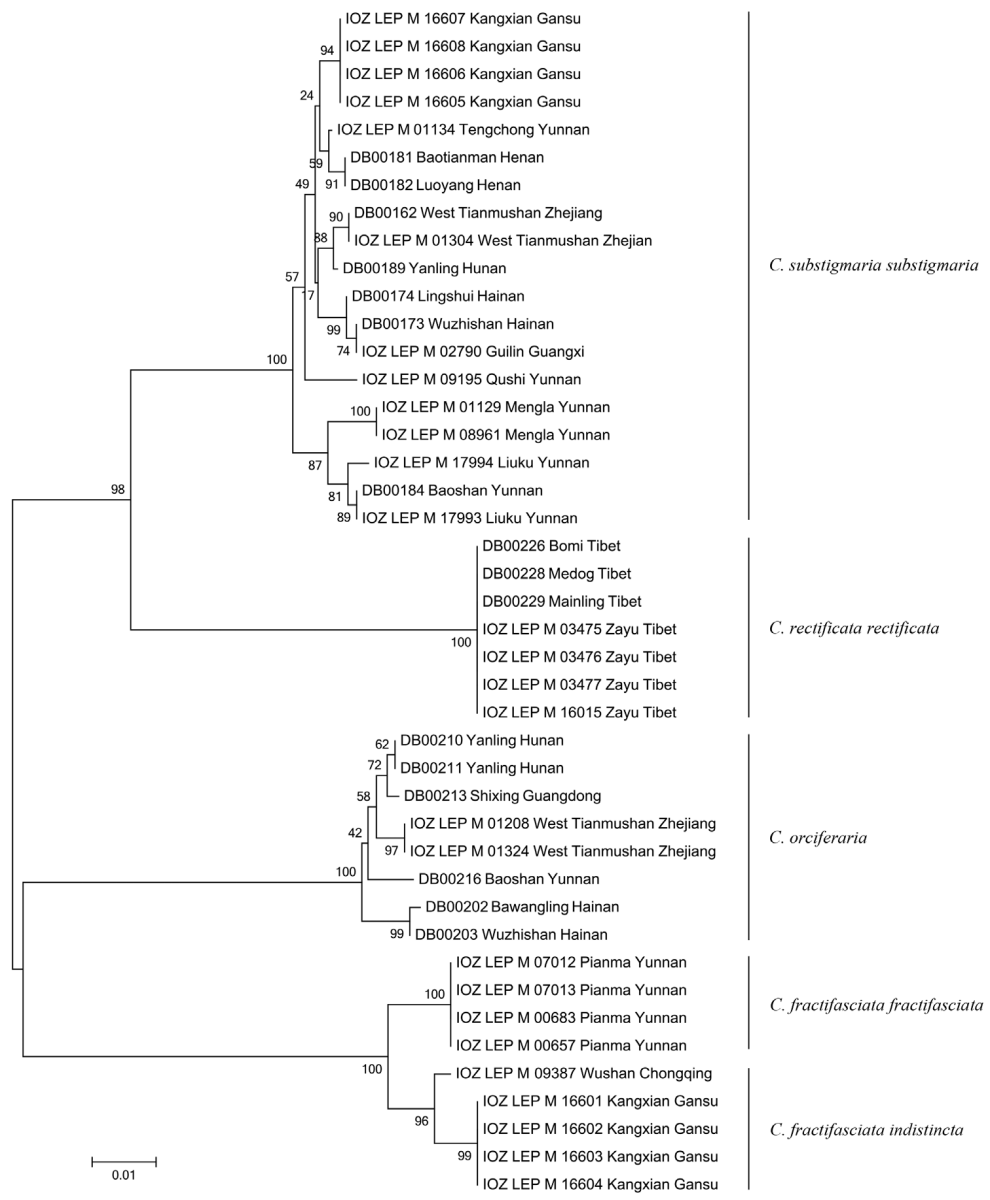
Neighbour joining tree (Kimura 2-parameter distance model for 658bp COI marker) for Chinese *Cyclidia* species. Terminals with sequence ID and collecting locality.

##### Distribution.

China (Yunnan, Tibet), India.

#### Cyclidia pitimani

Taxon classificationAnimaliaLepidopteraDrepanidae

(Moore, 1886)

Figs 12 , 13 , 31 , 42

Euchera pitimani Moore, 1886: 99. Syntypes including 1♂, Burma: Tenasserim, Tavoy (BMNH).
Cyclidia pitimani : Warren, 1922: 445.
Cyclidia sericea Warren sensu Chu & Wang, 1987: 206. (Misidentification)
Cyclidia sericea Warren sensu Chu & Wang, 1991: 64, fig. 24, pl. 1: 4. (Misidentification)

##### Diagnosis.

This species is very similar to *Cyclidia sericea* (Borneo, Sumatra), but can be distinguished by the following characters: smaller than *Cyclidia sericea* (the average forewing length of the male is 32 mm, against *ca* 40 mm in *Cyclidia sericea*); in *Cyclidia pitimani*, the doubled antemedial line form almost right angles anteriorly, especially the inner line, while in *Cyclidia sericea*, the protrusions of the antemedial lines are more rounded; the anterior part of the median band is much narrower in *Cyclidia pitimani*; the terminal spots are less distinct than those of *Cyclidia sericea*. In the male genitalia, the terminal part of the valva is broader and more rounded.

##### Material examined.

**CHINA: Yunnan** (IZCAS): 2♂, Xishuangbanna, Xiaomengyang, 850 m, 6–7.IX.1957, coll. Zang Lingchao and Zhang Yiran (one male was originally incorrectly recorded as “Qinghai, Gonghe”); 2♂, Xishuangbanna, Bubang, 700 m, 14.IX.1993, coll. Yang Longlong.

##### Genetic data.

No genetic data available.

##### Remarks.

After examining the types of *Cyclidia pitimani* and *Cyclidia sericea*, and studying the descriptions and figures of the two species (Moore 1886, Warren 1922, Holloway 1998, Chen 2011), we found that the specimens from Yunnan which were identified as *Cyclidia sericea* by Chu and Wang (1987, 1991) well agree with *Cyclidia pitimani*. Thus, *Cyclidia sericea* in Chu and Wang (1987, 1991) is considered to be a misidentification of *Cyclidia pitimani*.

Chu and Wang (1987, 1991) recorded one male specimen from “Qinghai, Gonghe, 3150 m, 6.IX.1957, coll. Zang Lingchao”. After examination, it was noted that the locality on the label of this specimen was incorrect. According to the collecting records of IZCAS, the collector (Zang Lingchao) went to Xiaomengyang of Xishuangbanna in Yunnan on September 6th, 1957, and no collector went to Qinghai on that date. We also found another specimen of *Cyclidia pitimani* which was collected at the same locality on September 7th, 1957. So, the locality on label should be written as Yunnan, Xishuangbanna, Xiaomengyang, 850 m. Qinghai should be deleted from the range area of *Cyclidia sericea* and the species should be deleted from the fauna of China.

##### Distribution.

China (Yunnan), Myanmar.

#### Cyclidia fractifasciata

Taxon classificationAnimaliaLepidopteraDrepanidae

(Leech, 1898)

Euchera fractifasciata Leech, 1898: 360. Syntypes 1♂, 1♀, China: Western China (BMNH).
Cyclidia fractifasciata : Gaede 1931: 2.

##### Diagnosis.

The species can be distinguished by the following characters: a black broad subbasal line is present on the forewing; the forewing medial line is broad at anterior half and very narrow and dot-like at posterior half; outer margin of the forewing medial line forms an almost right angle below M_3_; the phallus of the male genitalia forms a small protrusion posteriorly; the lamella postvaginalis of the female genitalia is rectangle.

##### Remarks.

Chu and Wang (1991) did not record this species. The specimens from Yunnan should be identified as *Cyclidia fractifasciata fractifasciata*, and the specimens from Gansu and Chongqing should be identified as a new subspecies, *Cyclidia fractifasciata indistincta* subsp. n., based on adult morphology and DNA barcodes.

##### Distribution.

China.

#### Cyclidia fractifasciata fractifasciata

Taxon classificationAnimaliaLepidopteraDrepanidae

(Leech, 1898)

Figs 14 , 15 , 32 , 43 , 53

##### Diagnosis.

See under *Cyclidia fractifasciata indistincta*.

##### Material examined.

**CHINA: Yunnan** (IZCAS): 1♂, Dulongjiang, 1500 m, 29.V.2006, coll. Xiao Ningnian; 3♂1♀, Lushui, Pianma, 8–12.V.2011, coll. Yang Xiushuai and Wang Ke.

##### Distribution.

China (Yunnan).

##### Genetic data.

The intrasubspecific divergence of the barcode region in *Cyclidia fractifasciata fractifasciata* is 0% (n = 4).

#### Cyclidia fractifasciata indistincta

Taxon classificationAnimaliaLepidopteraDrepanidae

Jiang, Han & Xue subsp. n.

http://zoobank.org/8BC2D3BA-389A-43E9-991E-CA93B3DE1837

Figs 16 , 17 , 33 , 44 , 54

##### Description.

***Head*.** Antennae blackish brown dorsally, flat and unipectinate, basal half without rami, rami very short. Frons blackish grey, not protruding. Labial palpi black with third segment distinct, extending beyond frons. Vertex black scattered with grey scales.

***Thorax*.** Patagia white at basal half and blackish grey at terminal half. Tegula blackish grey. Dorsal side of thorax white with two pairs of blackish grey patches medially. Hind tibia with two pairs of spurs in both sexes. Forewing length: 37–40 mm. Apex of forewing rounded, not falcate; outer margin of both wings smooth. Wings white, transverse lines black. Forewing with a blackish brown patch basally; subbasal line broad; antemedial lines double, outer line indistinct and often invisible; medial line broad band-like at anterior half, very narrow and dot-like at posterior half; outer margin of medial line forming an almost right angle below M_3_; discal spot white, almost rhombic; postmedial lines double, wavy, inner line very obscure; submarginal line double, broad, and invisible between M_3_ and CuA_1_; terminal lines double and discontinuous on each vein, inner line composed of oval markings, outer line appearing as series of short strips, inner markings often fused with outer ones; fringes white mixed with blackish grey. Hind wing with indistinct submarginal line; terminal lines and fringes similar to those of forewing. Underside white, striations indistinct than those of upperside.

***Abdomen*.** Abdominal segments diffused with white scales. Pairs of black quadrate markings on first to seventh abdominal segments. Anterotergal syndeses developed at anterior margin of 2^nd^ tergum. A pair of androconial hair-pencils present on 2^nd^ pleuron of male.

***Male genitalia*.** Uncus triangular. Socii sclerotized, about four-fifths the length of uncus. Gnathos with median process small and triangular. Valva narrow terminally; costa sclerotized and almost straight. Juxta formed a pair of forcipiform processes posteriorly. Saccus semicircular, about two-fifths length of basal width. Phallus slightly curved, with a small triangular lateral process posteriorly; vesica without cornuti.

***Female genitalia*.** Lamella postvaginalis rectangle. Ductus bursae with a colliculum, long and narrow, striate longitudinally. Corpus bursae oval, with a paired slender signa; signa separated and parallel.

##### Diagnosis.

The subspecies is very similar to the nominate subspecies, but differs externally by the following characters: the outer line of the antemedial line and the inner line of the postmedial line on the forewing are invisible, while in the nominate subspecies, they are much more distinct; the forewing discal spot is larger; the inner terminal markings of the forewing are larger and fused with the outer ones partly, while in *Cyclidia fractifasciata fractifasciata*, they are often smaller and separated from the outer ones.

##### Type material examined.

Holotype, ♂, **CHINA: Gansu** (IZCAS): Kangxian, Yangba, Meiyuangou, 1000 m, 13.VIII.2014, coll. Xue Dayong and Ban Xiaoshuang. Paratypes: 3♂2♀, same data as holotype. **Chongqing** (IZCAS): 1♀, Wushan, Wulipo, Dangyang, Congping, 1773 m, 25.VII.2013, coll. Cheng Rui.

##### Genetic data.

The intrasubspecific divergence of the barcode region in *Cyclidia fractifasciata indistincta* is 1%. The intraspecific divergence of the barcode region between *Cyclidia fractifasciata fractifasciata* (n = 4) and *Cyclidia fractifasciata indistincta* (n = 5) is 2.3%. The distance between *Cyclidia fractifasciata* with the nearest neighbour species *Cyclidia substigmaria* is 12.5%.

##### Distribution.

China (Gansu, Chongqing).

##### Etymology.

The subspecies is named on the basis of the Latin adjective *indistinctus*, referring to the transverse lines of the forewing.

#### Cyclidia orciferaria

Taxon classificationAnimaliaLepidopteraDrepanidae

Walker, 1860

Figs 18 , 19 , 34 , 45 , 55

Cyclidia orciferaria Walker, 1860: 56. Syntypes, China: North China.
Cyclidia ociferaria Kirby, 1892: 725. [Incorrect spelling of *Cyclidia orciferaria* Walker.]

##### Diagnosis.

This species is different from other congeners in the following external characters: the apex of the forewing is falcate; the wing colour is blackish brown; two bands covered with greyish blue scales are present on the forewing, and the inner band is narrower and less distinct than the outer band; the discal spot of the forewing is yellowish brown, oblong, with a blackish brown narrow line medially; greyish blue scales are covered on the submarginal lines of both wings, and often absent on the middle part of the hind wing. There are also differences in the male genitalia: the socii are weakly sclerotized and much shorter than the uncus; the valva is short. In the female genitalia, the posterior margin of the lamella postvaginalis is slightly concaved; the two signa are tapered at posterior half and situated very close to each other.

##### Material examined.

**CHINA: Zhejiang** (IZCAS): 2♂, Tianmushan, 20–23.VII.1973, coll. Zhang Baolin; 1♂1♀, Lin’an, West Tianmushan, 400–1500 m, 26.VII–29.VIII.2003, coll. Xue Dayong et al.; 1♂, West Tianmushan, Zhonglieci, 363 m, 24.VII.2011, coll. Yan Keji; 1♂, West Tianmushan, Xianrending, 1506 m, 27.VII.2011, coll. Yan Keji; 2♂2♀, Taishun, Wuyanling, Shuangkengkou, 680 m, 28–29.VII.2005, coll. Lang Songyun; 1♀, Taishun, Siqianzhen, 250 m, 4.VIII.2005, coll. Lang Songyun; 1♂, Ningbo, V.1981. **Jiangxi** (IZCAS): 1♀, Huzhi, 28.VII.1990. **Hunan** (IZCAS): 2♂, Yanling, Taoyuandong, 631 m, 4–8.VII.2008, coll. Chen Fuqiang; 1♂, Tianpingshan, 25.VI.1981. **Fujian** (IZCAS): 1♂, Jiangle, Longqishan, 800 m, 15.IX.1990, coll. Yang Bin; 8♂, Wuyishan, 24.IV–21.V.1983, coll. Wang Linyao; 1♀, Wuyishan, Sangang, 24.VII.1980; 1♀, Nanping, Shangyang, 9.VI.1963, coll. Zhang Youwei. **Guangdong** (IZCAS): 1♂, Ruyuan, Nanling, Baohuzhan, 1020 m, 16–20.VII.2008, coll. Chen Fuqiang; 1♀, Shixing, Chebaling, 365–401 m, 22–26.VII.2008, coll. Chen Fuqiang. **Hainan** (IZCAS): 4♂2♀, Nankai, Nanmaola, 1261 m, 10–14.V.2009, coll. Chen Fuqiang and Yan Keji; 6♂1♀, Jianfengling, Tianchi, 828 m, 1–5.V.2007, 18.V.2009, coll. Chen Fuqiang; 1♂2♀, Bawangling, Dong’er Linchang, 1004–1015 m, 8.V.2007, 7.IV.2008, coll. Chen Fuqiang and Lang Songyun; 11♂, Wuzhishan, Shuiman, 730–900 m, 7–11.V.2007, 1–3.IV.2008, coll. Lang Songyun and Han Hongxiang; 1♂3♀, Lingshui, Diaoluoshan, 190–920 m, 3–7.V.2007, coll. Han Hongxiang and Lang Songyun; 1♀, Qiongzhong, Limuling, 620 m, 15.V.2007, coll. Han Hongxiang; 1♀, Xinglong, 24.IV.1963, coll. Zhang Baolin. **Guangxi** (IZCAS): 3♂2♀, Fangcheng, Fulong, 200–550 m, 23–26.V.1999, coll. Yuan Decheng et al.; 1♂1♀, Napo, Defu, 1350 m, 19.VI.2000, coll. Zhu Chaodong; 1♂, Jinxiu, Linhai Shanzhuang, 1100 m, 2.VII.2000, coll. Li Wenzhu; 1♂, Jinxiu, Jinzhong Gonglu, 1000 m, 10.V.1999, coll. Han Hongxiang; 1♀, Daxin, Xialei, 680 m, 31.III.1998, coll. Li Wenzhu. **Yunnan** (IZCAS): 1♂1♀, Hekou, Xiaonanxi, 200 m, 10–11.VI.1956, coll. Huang Keren et al.; 1♀, Pingbian, Daweishan, 1500 m, 20.VI.1956, coll. Huang Keren et al.; 1♂, Xishuangbanna, Mengla, Menglun, 650 m, 1.VI.1964, coll. Zhang Baolin; 1♂1♀, Mengla Linchang, 550 m, 20.IV.1982, coll. Wang Yongxian; 1♂, Mengla, 20.VI.1982, coll. Chen Yixin; 1♀, Mengla, Lengku, 623 m, 10.VI.1980, coll. Guo Zuyun; 1♂, Xishuangbanna, Bubang, 700 m, 14.IX.1993, coll. Yang Longlong; 1♂, Xishuangbanna, Damenglong, 650 m, 1.VIII.1958, coll. Zheng Leyi; 1♂, Xishuangbanna, Dameng’a, 1050–1080m, 15.VIII.1958, coll. Wang Shuyong; 2♂, Cangyuan, 790–1100 m, 19–22.V.1980, coll. Song Shimei and Shang Jinwen; 1♂, Xiaomenglun, 21.IV.1982, coll. Wang Linyao; 1♂, Ruili, Dengga, 6–8.VI.1992, coll. Xue Dayong; 1♂, Baoshan, Baihualing, 1520 m, 11–13.VIII.2007, coll. Wu Chunguang.

##### Genetic data.

The intraspecific divergence of the barcode region of *Cyclidia orciferaria* is ranges from 0%–1.7% (average distance 1.09%) (n = 8). The distance to the nearest neighbour *Cyclidia substigmaria* is 11.5%.

##### Distribution.

China (Jiangsu, Zhejiang, Jiangxi, Hunan, Fujian, Guangdong, Hainan, Guangxi, Sichuan, Yunnan), Myanmar, Vietnam, Indonesia.

##### Biological notes.

The morphology of the larva of *Cyclidia orciferaria* was illustrated in Chen (2011).

#### Mimozethes

Taxon classificationAnimaliaLepidopteraDrepanidae

Warren, 1901

Mimozethes Warren, 1901: 190. Type species: *Euchera nana* Warren, 1897, by original designation.

##### Generic characters.

***Head*.** Antennae lamellate and shortly unipectinate, basal part of antennae without rami (Fig. 1b). Frons not protruding. Labial palpi with second segment slightly curved, third segment oval. ***Thorax*.** Hind tibia with two pairs of spurs. Apex of forewing falcate; outer margin of forewing protruding. Wing. Wings colour dark brown. Forewing with silver grey antemedial line, sometimes indistinct; discal spot black and small; postmedial line silver grey, forming a “>” shaped protrusion near R_5_. Hind wing with medial line and postmedial line silver grey and almost straight. Black brown patches present near anal angle of both wings. Terminal lines of both wings composed of a series of blackish brown strips covering silver grey scales, very distinct towards apex. Underside with distinct discal spot, costa, apex and outer margin suffused with pale yellowish brown scales. Vein (Fig. 3b). Forewing with R_1_ separate, R_2–4_ and R_5_ stalked, M_2_ arising from middle of discocellulars; Hind wing with Sc+R_1_ close to Rs beyond distal cell, then far from Rs, M_2_ arising from middle of discocellulars. Anterotergal syndeses developed at anterior margin of 2^nd^ tergum (Fig. 2). A pair of androconial hair-pencils present on 2^nd^ sternum of male (Fig. 2). ***Male genitalia*.** Uncus triangular, acute terminally; socii undeveloped; gnathos connected at middle and with median process small and acute apically; sacculus forming a long process; juxta short and broad, concaved posteriorly; saccus broad and rounded terminally; Phallus short; vesica without cornuti. ***Female genitalia*.** Papillae anales broad and rounded; lamella postvaginalis large and oval, with many tiny spines; ductus bursae long and narrow, with a colliculum; corpus bursae oval, without a signum.

##### Diagnosis.

See under *Cyclidia*.

##### Remarks.

According to Inoue (1962), *Mimozethes argentilinearia* (Leech, 1897) occurs in Japan and Taiwan. However, it has not been recorded from Taiwan in later studies (Inoue 1992, Yan et al. 2009, Chen 2011). Thus, following that, we do not include the species in this paper.

##### Distribution.

China, Japan.

##### Key to Chinese *Mimozethes* species

**Table:** 

1	Outer margin of forewing weakly protruding; ventral margin of valva forming a small triangular protrusion apically in male genitalia	***Mimozethes angula***, Figs 20–21
–	Outer margin of forewing strongly protruding; ventral margin of valva not forming a small triangular protrusion apically in male genitalia	***Mimozethes lilacinaria***, Figs 22–23

#### Mimozethes angula

Taxon classificationAnimaliaLepidopteraDrepanidae

Chu & Wang, 1987

Figs 20 , 21 , 35 , 46 , 56

Mimozethes angula Chu & Wang, 1987: 207. Holotype ♂, China: Sichuan: Mt. Emei (IZCAS).

##### Diagnosis.

This species is very similar to *Mimozethes lilacinaria* (Leech, 1897) and *Mimozethes argentilinearia*, but it can be distinguished by the following characters: the outer margin of the forewing is less strongly protruding than that of *Mimozethes lilacinaria* and *Mimozethes argentilinearia*; the black patch inside the anal angle of the forewing is less distinct than that of *Mimozethes argentilinearia*; the yellowish brown patch on the underside of the forewing is much smaller and less distinct than that of *Mimozethes lilacinaria* and *Mimozethes argentilinearia*. In the male genitalia, the uncus is shorter; the ventral margin of the valva forms a small triangular protrusion apically, but *Mimozethes lilacinaria* and *Mimozethes argentilinearia* lack this character; the sacculus process is much longer than that of *Mimozethes lilacinaria*.

##### Type material examined.

**CHINA: Sichuan** (IZCAS): 1♂ (Holotype), Emeishan, Qingyinge, 800–1000 m, 15.IX.1957, coll. Zhu Fuxing; 1♀ (Allotype), same locality, 22.IX.1957, coll. Zhu Fuxing; 4♂2♀ (Paratype), same locality, 22.VI.1957, 15–19. IX.1957, coll. Zhu Fuxing *et al*.

##### Additional material examined.

**CHINA: Henan** (IZCAS): 1♀, Baiyunshan, 13–15.VIII.2008, 1550 m, coll. Jiang Nan. **Hubei** (IZCAS): 1♂, Shennongjia, Dajiuhu, 1800 m, 1.VIII.1981, coll. Han Yingheng. **Sichuan** (IZCAS): 9♂2♀, Emeishan, Qingyinge, 800–1000 m, 20.VI.1957, 15–22.IX.1957, coll. Zhu Fuxing et al.; 1♀, Qingchengshan, 1000 m, 4.VI.1979, coll. Shang Jinwen; 1♂, Emeishan, 1288 m, 31.VII.2013, coll. Cheng Rui.

##### Genetic data.

No genetic data available.

##### Distribution.

China (Henan, Hubei, Sichuan).

#### Mimozethes lilacinaria

Taxon classificationAnimaliaLepidopteraDrepanidae

(Leech, 1897)

Figs 22 , 23 , 36 , 47 , 57

Decetia lilacinaria Leech, 1897: 184. Holotype ♂, China: Sichuan: Emeishan (BMNH).
Heteromize lycoraearia Oberthür, 1912: 269. Holotype ♂, China: Sichuan: Mou-pin (BMNH).
Mimozethes lilacinaria : Beccaloni et al. 2003 [accessed 26 November 2015].

##### Diagnosis.

See under *Mimozethes angula*.

##### Type material examined.

**CHINA: Sichuan** (BMNH): 1♂ (Holotype), Omei-Shan, 3620 ft., Native coll. July & Aug. 1890, Leech Coll. 1900-64, BMNH (E) 1377104.

##### Additional material examined.

**CHINA: Sichuan** (BMNH): 1♂, Chasseurs indigènes, de Tà-tsien-lou, Récolle de 1910, Ex Oberthür Coll. Brit. Mus. 1927-3, Drepanidae genitalia slide No. 304; 1♀, Siao-Lou, 1900, Chasseurs indigènes, Ex Oberthür Coll. Brit. Mus. 1927-3. **Yunnan** (IZCAS): 1♀, Xishuangbanna, Menghai, 21.VII.1958, coll. Wang Shuyong.

##### Genetic data.

No genetic data available.

##### Remarks.

Chu and Wang (1991) did not record this species. The specimens from Yunnan should be identified as *Mimozethes lilacinaria* based on adult morphology.

##### Distribution.

China (Sichuan, Yunnan).

### DNA barcoding results and discussion

Forty-three DNA barcode sequences of lengths 658bp were obtained for *Cyclidia* species. The nucleotide composition of *Cyclidia* species COI genes was 30.60% of A, 38.54 of T, 16.06% of C, 14.80% of G. The interspecific distance within the genus was range from 8.8%–13.9%. The maximum intraspecific distances was 2.6% in *Cyclidia substigmaria*, 1.7% in *Cyclidia orciferaria*, 0.0% in *Cyclidia rectificata*, and 2.3% in *Cyclidia fractifasciata*. The maximum genetic distances observed within species (2.6% at COI) were less than the minimum distances observed between the species (8.8%). There is a clear barcoding gap between intra and interspecific variation; furthermore, NJ tree also provided strong support for the separation of *Cyclidia* species (Fig. 58).

In recent revisionary work of Drepanidae, Song et al. (2011, 2012) and Park et al. (2011) found many new taxa, synonyms and misidentifications in earlier studies. However, when dealing with some morphologically similar taxa, it is difficult to discriminate only using the subtle diagnostic characters. The present study utilizing morphological and molecular characters revised some Chinese *Cyclidia* species. The morphological analysis indicated that some structures of the genitalia were found to be less diagnostic than the external characters between some species (i.e. *Cyclidia substigmaria* and *Cyclidia rectificata*). Sihvonen et al. (2014) also mentioned this trait in the Geometridae. Additionally, some structures of the male genitalia (e.g. the shape of the valva) sometimes varied among individuals of *Cyclidia substigmaria*. Therefore, species have been delineated on the basis of a combination of data from morphology and DNA barcodes. In the molecular analysis, DNA barcodes proved to be very helpful. The interspecific divergence of *Cyclidia* species (minimum distance 8.8%, maximum distance 13.9%) was much larger than the 2% or 3% of the threshold for species diagnosis (Hebert et al. 2003, Hebert et al. 2004a, Hebert et al. 2004b). The remarkably high interspecific divergence and low intraspecific divergence on average 1% (minimum distance 0.0%, maximum distance 2.6%) fully supports the morphological species concept.

## Supplementary Material

XML Treatment for Cyclidia

XML Treatment for Cyclidia substigmaria

XML Treatment for Cyclidia substigmaria substigmaria

XML Treatment for Cyclidia substigmaria intermedia

XML Treatment for Cyclidia rectificata

XML Treatment for Cyclidia rectificata rectificata

XML Treatment for Cyclidia pitimani

XML Treatment for Cyclidia fractifasciata

XML Treatment for Cyclidia fractifasciata fractifasciata

XML Treatment for Cyclidia fractifasciata indistincta

XML Treatment for Cyclidia orciferaria

XML Treatment for Mimozethes

XML Treatment for Mimozethes angula

XML Treatment for Mimozethes lilacinaria
